# Breast Cancer Data Analysis Using Supervised Machine Learning Algorithms

**DOI:** 10.7759/cureus.95011

**Published:** 2025-10-20

**Authors:** Durga H Kutal, Beyza N Koseoglu

**Affiliations:** 1 Mathematics, Augusta University, Augusta, USA

**Keywords:** breast cancer dataset, decision tree, logistic regression, machine learning, random forests, support vector machines

## Abstract

Breast cancer is one of the most serious diseases and a leading cause of cancer-related deaths for women worldwide. This study evaluates and compares the performance of several supervised machine learning algorithms for breast cancer tumor classification, using a real-world dataset (sourced from Kaggle.com). From an initial 212 observations, the final dataset was reduced to 205 after handling missing values. We employed logistic regression, decision tree, random forest, and support vector machines (SVMs) with various kernels, focusing on model accuracy, feature importance, and the impact of dimensionality reduction. All models demonstrated strong performance, with accuracies above 87%. The most effective classifiers were the random forest and polynomial SVM, achieving the highest area under the curve (AUC) values of 96.3% and 96.9%, respectively. Feature importance analysis consistently identified Tumor Size, Involved Lymph Nodes, Metastasis, and Age as the most significant predictors. The high accuracy of simpler models, such as logistic regression and a linear SVM, is attributed to the dataset's inherent linear separability. Our findings also validate the use of principal component analysis (PCA) for feature reduction, as key models maintained high performance on the simplified dataset.

## Introduction

Breast cancer remains one of the most common malignancies and a leading cause of cancer-related death among women globally. According to the World Health Organization (WHO), early detection and accurate diagnosis significantly improve treatment outcomes and survival rates. While traditional diagnostic methods such as mammography, histopathological analysis, and biopsies are standard practice, they often suffer from limitations, including subjectivity, time constraints, and diagnostic variability among clinicians. Artificial intelligence (AI) applications in oncology, leveraging comprehensive datasets, include risk assessment, early diagnosis, patient prognosis estimation, and treatment selection. Machine learning (ML), a subset of AI that enables computers to learn from training data, has been highly effective at predicting various types of cancer, including breast, brain, lung, liver, and prostate cancer [1]. These challenges have led to the growing integration of ML techniques into clinical decision support systems.

Supervised ML algorithms, in particular, have been widely used for binary classification tasks such as distinguishing between benign and malignant tumors. These algorithms learn patterns from labeled data and are capable of handling complex relationships within high-dimensional clinical datasets [2,3]. Recent studies have demonstrated high accuracy using models like support vector machines (SVMs), random forest, decision tree, and logistic regression for breast cancer prediction. SVM, in particular, has been noted for its ability to separate classes effectively using different kernel functions, especially in medical imaging and classification problems.

This study applies these algorithms to a real-world breast cancer dataset (sourced from Kaggle.com) that originally contained 213 patient records collected between 2019 and 2020. La Moglia and Almustafa [4] previously analyzed this dataset, demonstrating the effectiveness of multiple models. In our study, we applied a more rigorous data cleaning process: excluding incomplete records, consolidating breast quadrant categories, and removing non-predictive identifiers such as "S/N" and "year". This resulted in a final dataset of 205 observations and nine variables. We examine several supervised ML methods, assess the impact of different SVM kernels, and apply dimensionality reduction to gain deeper insights into the dataset's underlying structure and enhance interpretability. Each algorithm is trained and evaluated on this dataset using multiple performance metrics, including accuracy, sensitivity, specificity, F1 score, and area under the receiver operating characteristic (ROC) curve (AUC).

The primary objectives of this study are to (i) evaluate and compare the performance of various supervised ML algorithms for the classification of breast cancer tumors, (ii) systematically examine the impact of different SVM kernels on classification accuracy, (iii) assess the effect of principal component analysis (PCA) as a dimensionality reduction technique on model performance, and (iv) identify the most suitable algorithm that provides a balance between high predictive accuracy and clinical interpretability.

This study conducts a comparative analysis of four widely applied supervised ML models, such as logistic regression, decision tree, random forest, and SVMs, with different kernel functions for breast cancer classification. These models were selected to represent a balance between interpretability and predictive power: Logistic regression, a statistical approach to predict the presence of a disease based on available variables (symptoms, imaging data, patient history, etc.), has been successfully used for prediction and diagnosis in medicine [5]. Decision tree offers intuitive rule-based classification [6]. Random forest employs many different classification techniques; every one of them may be put into action using a decision tree. The use of multiple decision trees improves classification accuracy and enhances model robustness [7]. SVM is well-suited for handling high-dimensional data. For the SVM classifier, four kernel functions, namely, linear, polynomial, radial basis function (RBF), and sigmoid, were further evaluated to assess their ability to capture both linear and nonlinear patterns, a method often employed to enhance classification in complex medical datasets [8].

## Materials and methods

Preparing the dataset

The dataset for this study was sourced from Kaggle.com and was originally compiled by Fatemeh Mehrparvar [9]. The initial dataset contained 213 observations. To maintain analytical integrity, one record missing all attribute values and seven records with missing values were excluded from this study. The final, cleaned dataset used for this analysis consists of 205 records. The "S/N" and "year" attributes were removed from the dataset prior to analysis, as they are identifiers and not relevant for predicting the diagnostic outcome. The dataset included eight predictor variables and a single response variable used for analysis.

The predictor variables consisted of both continuous and categorical measures, encompassing clinical and demographic features. The features are as follows: patient age at the time of diagnosis (Age), Tumor Size (cm), axillary lymph node status (Inv. Nodes: 0=absent; 1=present), menopausal status (Menopause: 0=postmenopausal; 1=premenopausal), presence of metastasis (Metastasis: 0=no; 1=yes), tumor location within the breast (Breast Quadrant, encoded as 1 for lower inner, 2 for lower outer, 3 for upper inner, and 4 for upper outer), affected breast side (Breast: 0=left; 1=right), and history of cancer (History: 0=no; 1=yes). The outcome variable was the diagnosis result, encoded as 1 for malignant and 0 for benign. To prepare for modeling, the final dataset was partitioned into a training set, containing 80% of the records, and a testing set with the remaining 20%. All data processing, modeling, and analysis were performed using Python (Version 3.10.2, Python Software Foundation, Wilmington, Delaware, United States) and R (Version 4.3.2, R Foundation for Statistical Computing, Vienna, Austria).

ML models and classification methods

To analyze the dataset and classify the breast cancer tumors, we employed four supervised ML models: logistic regression [10], decision tree [11], random forest [12], and SVMs [13]. For the SVM classifier, we further investigated the performance of four different kernel functions, namely, linear, polynomial, RBF, and sigmoid, to evaluate their ability to capture both linear and nonlinear relationships within the data. The detailed implementation, specific parameter settings, and comparative performance of each of these models are presented and discussed in the Results section.

t-distributed stochastic neighbor embedding (t-SNE)

**Figure 1:**
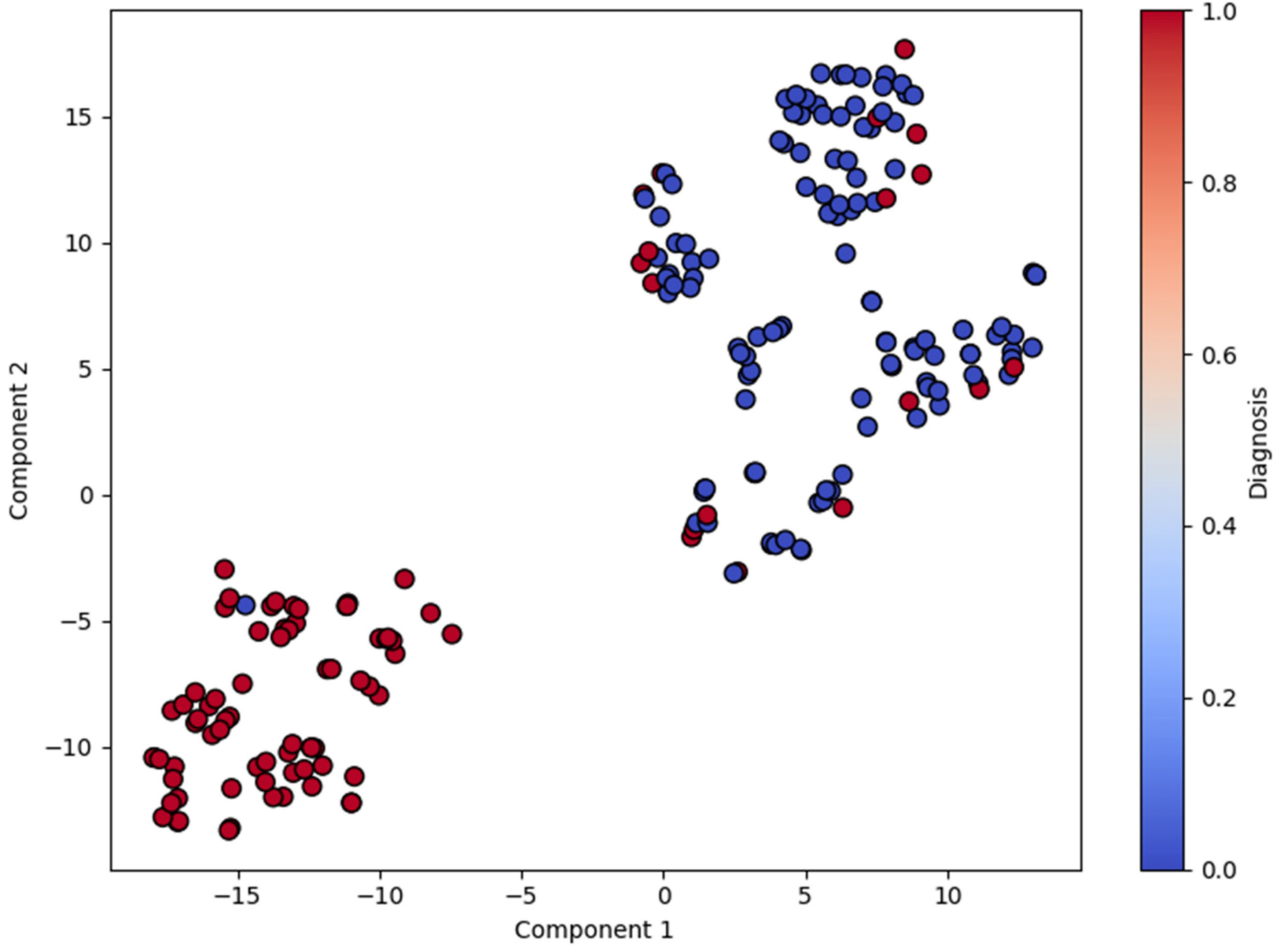
The t-SNE projection of the dataset showing separation between classes t-SNE: t-distributed stochastic neighbor embedding

The t-SNE plot (Figure 1) visualizes the high-dimensional breast cancer data into a simplified 2D space that reveals subtle, nonlinear clustering patterns [14]. The 2D t-SNE visualization clearly illustrates the separation between benign and malignant samples, offering a visual confirmation of the intrinsic structure within the dataset. This separation underscores the dataset's suitability for classification and highlights the value of using nonlinear methods alongside traditional linear techniques. The blue dots represent the benign class, and the red dots represent the malignant class. The red (malignant) points are tightly grouped together and completely separated from the blue (benign) points. The plot provides strong visual evidence that the two classes (benign and malignant) are inherently separable. This is a critical finding because it validates that the dataset contains a strong signal and is well-suited for a classification task. This suggests that the ML algorithm will be able to learn to accurately distinguish between benign and malignant tumors based on these features.

Model evaluation metrics

The performance of the classification models was assessed using the confusion matrix, which summarizes prediction outcomes into four categories: true positives (TP), true negatives (TN), false positives (FP), and false negatives (FN). These values provide the basis for several standard evaluation metrics that are widely applied in biomedical and ML research, namely, accuracy, sensitivity (recall), specificity, F1 score, and AUC.

The metrics were computed using the following equations [15]: $\text{Accuracy}\%=\frac{\text{TP+TN}}{\text{TP+TN+FP+FN}}\times100$, $\text{Sensitivity}\%=\frac{\text{TP}}{\text{TP+FN}}\times100$, $\text{Specificity}\%=\frac{\text{TN}}{\text{TN+FP}}\times100$, $\text{F1 Score}\%=\frac{2\times\text{TP}}{2\times\text{TP+FP+FN}}\times100$, and $\text{AUC} = \int_{0}^{1} \text{Sensitivity} \,d(1 - \text{Specificity})$.

Accuracy measures the overall proportion of correctly classified cases, while sensitivity evaluates the model's effectiveness in identifying TP. Specificity reflects the ability to correctly detect TN, and the F1 score provides a balanced measure that accounts for both precision and recall [16], making it especially useful in cases of class imbalance. Finally, AUC is a widely used metric that summarizes a model's diagnostic performance across all classification thresholds [17]. It signifies the probability that the model will correctly rank a randomly chosen malignant case higher than a randomly chosen benign case. AUC values range from 0.5 (equivalent to random chance) to 1.0 (perfect discrimination). For diagnostic studies, an AUC value above 0.90 is considered to indicate excellent performance, while a value below 0.80 suggests limited clinical usability, even if statistically significant [17].

Interpreting accuracy variations across R and Python

Although the same dataset and kernel types were used, small differences in classification accuracy were observed between the R and Python implementations. These differences are mainly due to variations in default settings for model parameters, how the algorithms are optimized internally, and possible differences in how the data was preprocessed (such as scaling). Such small differences are normal when using different software tools and do not mean that either one is incorrect. The performance comparison shown in Table 1, for example, is specific to the SVM models.

**Table 1:** SVM kernel accuracies between R and Python implementations RBF: radial basis function; SVM: support vector machine

Kernel	R accuracy (%)	Python accuracy (%)	Difference
Linear	90	90.62	≈ +0.62
Polynomial	90	87.5	≈ −2.5
RBF	87.5	89.06	≈ +1.56
Sigmoid	90	90.62	≈ +0.62

## Results

Logistic regression model

Logistic regression is a supervised ML algorithm used for binary classification (benign and malignant), where it predicts the probability of an outcome that has two possible classes (e.g., malignant=1 or benign=0). It works by applying the sigmoid (logistic) function to transform input values into a probability, which always falls between 0 and 1: $f(x) = \frac{1}{1 + e^{-x}}$.

Logistic regression models the chance of an outcome based on individual characteristics. Because the probability, p, must be between 0 and 1, the algorithm doesn't model p directly. Instead, it models the log odds of the outcome, which is given by $log\left(\frac{p}{1-p}\right)=\beta_0+\beta_1 X_1+\beta_2X_2+\dots+\beta_8X_8$, where p is the probability of a positive outcome (malignant, 1), 1-p is the probability of a negative outcome(benign, 0), and β1,β2,...,β8 are the coefficients of the predictors X1,X2,...,X8, respectively.

The odds of an event are calculated with the following formula: $\frac{p}{1-p}$.

A positive coefficient means that when the predictor's value increases, the log odds of breast cancer malignancy increase, too. In our study, we used this model to predict the breast cancer diagnosis: 1 for malignant and 0 for benign.

**Figure 2:**
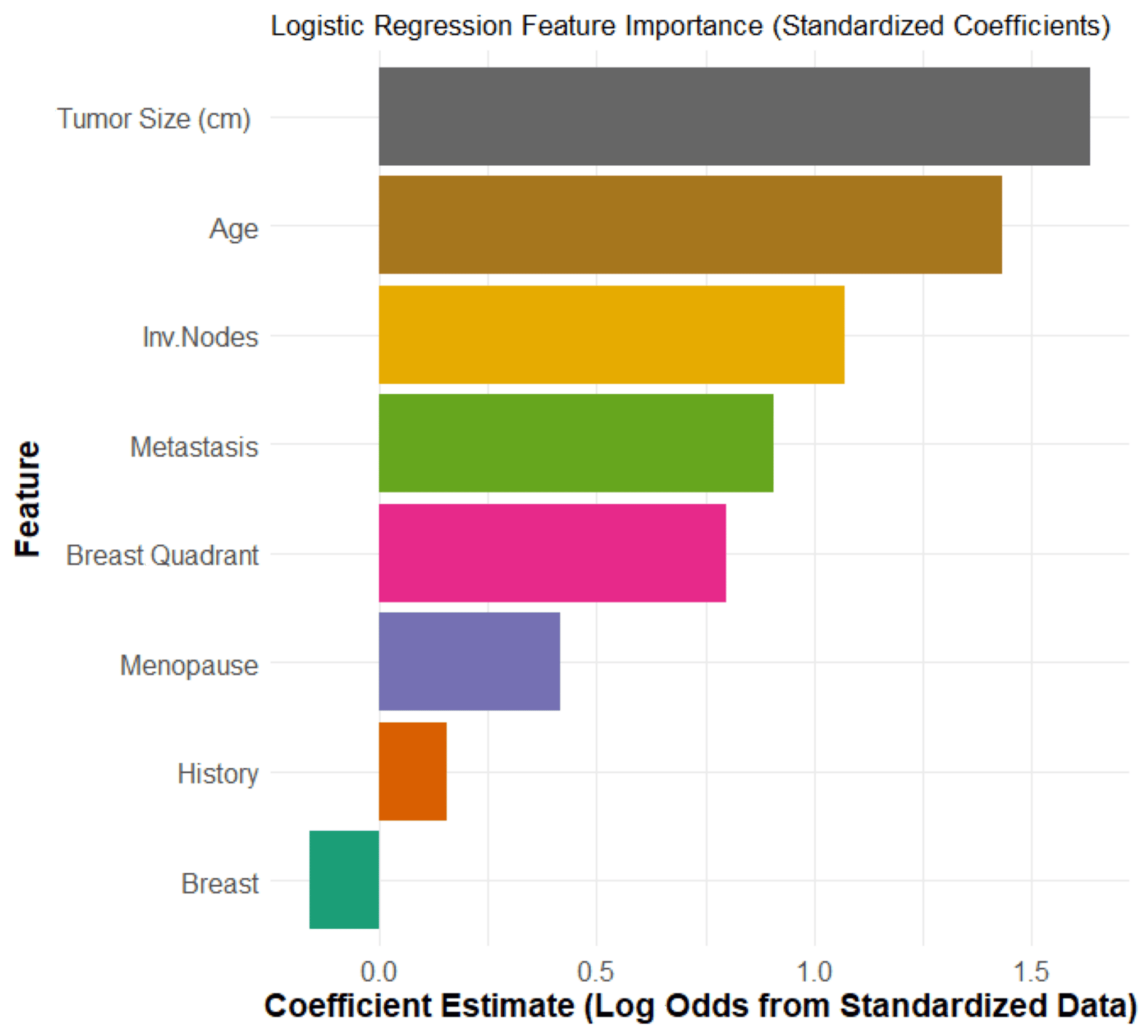
Logistic regression importance of coefficients Tumor Size: cm; Age: patient age at the time of diagnosis; Inv. Nodes: axillary lymph node involvement; Metastasis: presence of metastasis; Menopause: menopausal status; Breast Quadrant: tumor location within the breast; History: history of cancer; Breast: affected breast side

Figure 2 illustrates the magnitude of each feature's coefficient in the logistic regression model. Larger bars, positive or negative, indicate a stronger potential influence on the prediction. Based on the coefficient magnitudes, Tumor Size (cm) and Age are the most impactful predictors, followed by Involved Nodes and Metastasis.

However, a coefficient's magnitude must be interpreted alongside its statistical significance (p-value) to confirm its reliability. An analysis of the model's summary provides a detailed statistical interpretation. The features found to be statistically significant predictors of malignancy (with a p<0.05) are Tumor Size (cm) (p=0.0057), Age (p=0.009), and Breast Quadrant (p=0.025). An increase in the value of each of these features is associated with higher odds of a malignant diagnosis.

Several other features showed directional trends but were not statistically significant (p>0.05). Positive coefficients for Menopause (p=0.3244), Involved Nodes (p=0.1716), Metastasis (p=0.207), and History (p=0.625) suggest a potential increase in the odds of malignancy that did not reach statistical significance. Conversely, the Breast feature had a negative coefficient, suggesting lower odds for the right breast versus the left, but this finding was also not significant (p=0.634).

The ROC curve is a way to see how any predictive model can distinguish between the TP and TN. It characterizes classifier performance by plotting the TP rate (sensitivity) against the FP rate (1-specificity), where sensitivity reflects the probability of correctly identifying a positive instance and 1-specificity represents the probability of incorrectly classifying a negative instance as positive [18]. AUC is a frequently used summary measure of diagnostic/predictive accuracy [19]. It is calculated as 0.9386, suggesting that the model performs approximately 94% correctly in ranking a randomly chosen malignant tumor higher than a randomly chosen benign tumor.

Decision tree model

Decision tree is a foundational supervised ML algorithm, widely applied in classification tasks. In the context of this study, it was applied to classify breast cancer cases as malignant or benign [20]. The tree structure consists of a root node, internal nodes, branches, and leaf nodes. The root node represents the most important initial feature for splitting the dataset, the internal nodes represent the subsequent decision points, the branches connect the nodes, and the leaf nodes correspond to the final classification outcome [21].

In this article, the decision tree model was developed using the rpart package in R, which implements the Classification and Regression Trees (CART) algorithm. By default, the rpart package uses the GINI impurity criterion to evaluate splits. The Gini index measures the purity of the data at a node, with a lower value indicating a purer split where most samples belong to a single class, $Gini= 1 - \sum_{i=1}^{C} p_i^2$, where C is the number of classes. Since we have two classes, benign (0) and malignant (1), the Gini index is simplified to $Gini = 1 - (p_0^2 + p_1^2)$, where p_0_ is the proportion of class 0 (benign) and p_1_ is the proportion of class 1 (malignant). The process for selecting an optimal decision node using the Gini index involves a series of steps. First, the Gini index is calculated for each attribute. A weighted sum of Gini indices is then computed for the feature. The attribute with the lowest Gini index value is selected to be the decision node. This process is repeated for each subsequent node until the entire tree has been created [22].

Each leaf node represents the final classification of a tumor, providing classification (benign or malignant), probability (confidence in classification), and proportion of samples (percentage of total cases classified into this category). The first line (0 or 1) is the classification decision made at the leaf. The second line displays the percentage of all samples that fall into this leaf. The numbers shown above each split indicate the count of samples from the parent node that are directed down that particular branch.

**Figure 3:**
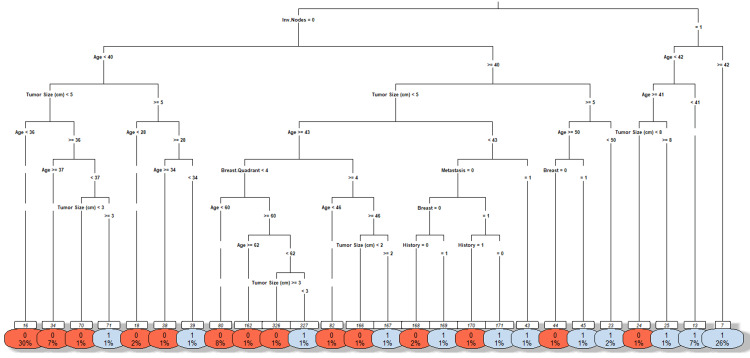
Decision tree model for breast cancer prediction Tumor Size: cm; Age: patient age at the time of diagnosis; Inv. Nodes: axillary lymph node involvement; Metastasis: presence of metastasis; Breast Quadrant: tumor location within the breast; History: history of cancer; Breast: affected breast side

Figure 3 illustrates the decision tree generated from the breast cancer dataset using the R statistical environment. The root node of this decision tree is the Involved Nodes variable (Inv. Nodes), indicating it is the most important initial discriminator for malignancy. If a patient has axillary lymph nodes, there is a high probability that the tumor is malignant. From there, the tree splits based on other factors, such as Tumor Size, Age, Breast Quadrant, Metastasis, History, and Breast (the affected breast side), before arriving at the leaf nodes. Tumor Size and Involved Nodes emerged as the most influential predictors, contributing significantly to the model's decision-making process. Age and Metastasis also emerged as moderately important predictors in the model. Variables such as Breast Quadrant, History, and Breast contributed less to the model, indicating weaker predictive power within this dataset analysis.

The decision tree model served as a solid baseline, achieving an accuracy of 0.875 and an AUC of 0.8849. This suggests that while a single, interpretable tree is useful, it is not the most powerful or accurate model for this classification task.

Random forest model

Random forest is a supervised ML algorithm widely used to analyze breast cancer datasets. It is a group of unpruned classification or regression trees made from the random selection of samples of the training data. The prediction is made by aggregating the predictions of the ensemble [23]. Random forest algorithm averages predictions over many individual decision trees. This process, known as bootstrap aggregating (or simply bagging), handles complex and high-dimensional data and helps reduce overfitting [24].

In this cancer study, the random forest model demonstrated strong performance, achieving an accuracy of 90% and a perfect specificity of 1.0000, but a slightly lower sensitivity of 76.47%, an F1 score of 86.67%, and an AUC of 96.55%. These results indicate an excellent ability to correctly identify malignant cases while maintaining high discrimination between malignant and benign samples.

**Figure 4:**
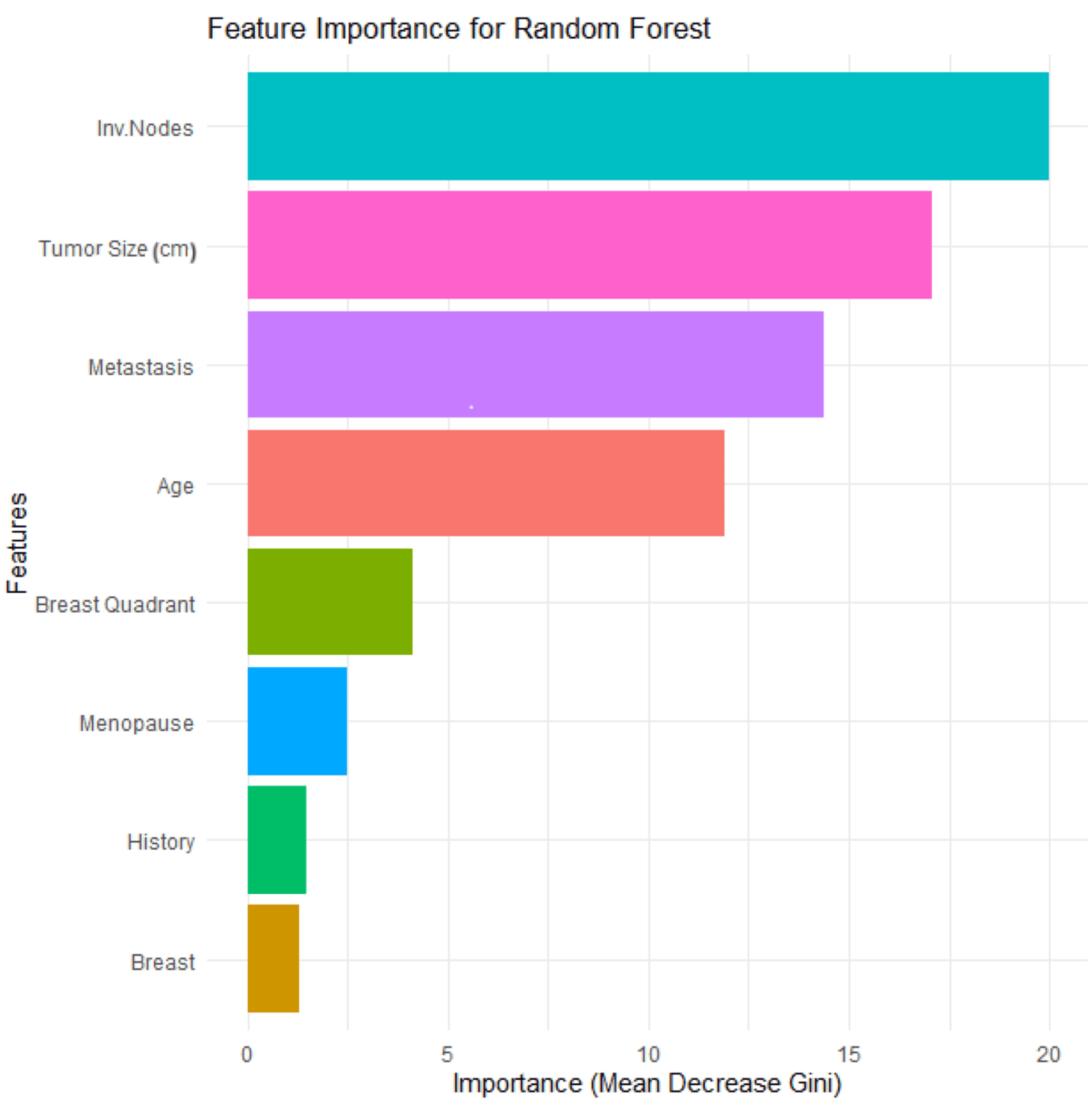
Feature importance rankings from the random forest model used to predict breast cancer diagnosis Tumor Size: cm; Age: patient age at the time of diagnosis; Inv. Nodes: axillary lymph node involvement; Metastasis: presence of metastasis; Menopause: menopausal status; Breast Quadrant: tumor location within the breast; History: history of cancer; Breast: affected breast side

The importance of the feature is calculated by adding up the improvement in the objective function given in the splitting criterion over all internal nodes of a tree and across all trees in the forest, separately for each predictor variable. The variable importance score is normalized by dividing all scores by the maximum score: the importance of the most important variable is always 100%. Feature importance analysis on this cancer data study revealed that Involved Lymph Nodes and Tumor Size were the most influential predictors in determining malignancy. Metastasis and Age were also important contributors, while features such as Breast Quadrant, Menopause, History, and Breast played a smaller role in the model's decisions. Random forests often perform much better on prediction tasks [24]. Figure 4 presents the variable importance rankings for the random forest model, highlighting the top predictors influencing breast cancer diagnosis in this dataset.

SVMs

SVMs are frequently used in breast cancer studies for classification tasks, such as distinguishing between malignant and benign tumors. In this study, SVMs aim to determine an optimal decision boundary that effectively separates benign from malignant tumors based on input clinical and demographic features [25]. This decision boundary, referred to as a hyperplane, is strategically constructed to maximize the margin between the two classes, benign and malignant. In a two-dimensional space, the hyperplane manifests as a line, while in higher dimensions, it generalizes to a plane or hyperplane. The hyperplane can be mathematically expressed by the equation $f (x) = w \cdot x + b$ [26] where w represents the weight vector orthogonal to the hyperplane, x denotes the input feature vector, and b is the bias term. The SVM optimization process seeks to identify the optimal values for w and b to maximize the margin while ensuring the accurate classification of the training data. Data points that lie precisely on the margin (i.e., where f(x) = ±1) are called support vectors.

For nonlinearly separable data, SVM employs the kernel trick, which maps inputs into a higher-dimensional feature space where linear separation becomes feasible [27]. Commonly used kernel functions include (i) linear kernel, (ii) RBF kernel, which maps points in a way that emphasizes their distance from one another, (iii) polynomial kernel, which introduces nonlinearity by considering feature combinations, and (iv) sigmoid kernel, which mimics the behavior of neural networks. We utilized SVM with these kernel functions to classify benign and malignant in this breast cancer data study.

PCA

PCA is a linear transformation technique that is used for dimensionality reduction. It works by reorienting the dataset into a new coordinate system defined by principal components (PCs) [28], which are a set of new, uncorrelated variables. These PCs are ordered so that the first PC captures the most variance in the data, the second PC captures the next most, and so on. The PCA process starts by centering the data (subtracting the mean from each feature): $X_{\text{centered}} = X_{ij} - \bar{X_j}$. Then, it computes the covariance matrix (Σ), which captures the relationships between features: $\Sigma=\frac{1}{n-1}X_{\text{centered}}^\top \cdot X_{\text{centered}}$. The directions of maximum variance are identified by solving the eigenvalue problem $\Sigma \cdot v = \lambda \cdot v$, where v are the eigenvectors (directions of the PCs) and λ are the eigenvalues (variance explained by each component). By selecting the top k eigenvectors associated with the largest eigenvalues, the reduced feature space is constructed as $Z = X_{\text{centered}} \cdot V_k$, where Vk is the matrix of the top k eigenvectors and Z is the transformed dataset.

In this study, we visualized the first two PCs (PC1 and PC2) and constructed a scree plot to assess the cumulative explained variance. Although PC1 and PC2 accounted for a significant portion of the variability of the data, more components were needed to exceed a 95% explained variance threshold. For this data analysis, we performed SVM classification on both the full dataset and a PCA-reduced version that retains at least 95% of the total variance.

SVM model performance and analysis

Following dimensionality reduction, we trained and evaluated SVM models using different kernel functions: linear, polynomial, RBF, and sigmoid. Each kernel was tested on both the full feature set and the reduced 2D input from PCA. The accuracy results are presented below in Table 2.

**Table 2:** SVM classification accuracy using the full feature set and first two principal components (PC1 and PC2) RBF: radial basis function; SVM: support vector machine

Kernel	Accuracy (full dataset)	Accuracy (PC1 and PC2)
Linear	90%	90%
Polynomial	90%	88.3%
RBF	87.5%	90%
Sigmoid	90%	73%

As shown in Table 2, comparison of classification accuracies on the full dataset indicates that the linear, polynomial, and sigmoid kernels each attained an accuracy of 90%, whereas the RBF kernel demonstrated a slightly lower accuracy of 87.5%. For the PCA-reduced dataset (PC1 and PC2), both the linear and RBF kernels achieved an accuracy of 90%, while the polynomial kernel yielded a slightly lower accuracy of 88.3%. In contrast, the sigmoid kernel showed a marked decline in performance, with accuracy dropping to 73%. These results suggest that the linear and RBF kernels are the most reliable classifiers for this dataset, maintaining high performance under both full and reduced feature representations.

Visualization of decision boundaries

**Figure 5:**
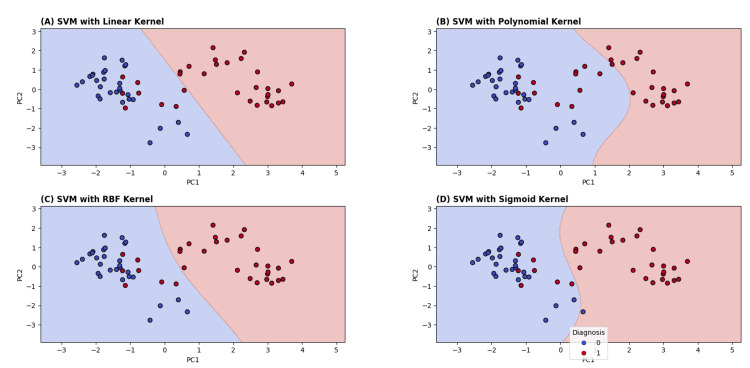
Decision boundary for each kernel function: (A) linear kernel, (B) polynomial kernel, (C) RBF kernel, and (D) sigmoid kernel RBF: radial basis function; SVM: support vector machine

The decision boundary plots for the four kernel functions provide visual insight into how each model separates benign and malignant cases in the two-dimensional PCA-transformed space. Figure 5 illustrates the decision boundaries generated by SVM classifiers with linear, polynomial, RBF, and sigmoid kernels, using the first two PCs (PC1 and PC2) as input features. Each plot shows how the respective kernel separates benign (blue) and malignant (red) breast cancer samples.

The decision boundary visualizations demonstrate that the linear and RBF kernels provide the most effective separation of benign and malignant samples, both quantitatively (highest accuracies) and qualitatively (clear and stable boundaries). The polynomial kernel, while flexible, shows signs of overfitting, and the sigmoid kernel demonstrates instability under dimensionality reduction. These results suggest that the dataset is effectively separable using either a linear or smooth nonlinear boundary.

The scree plot

**Figure 6:**
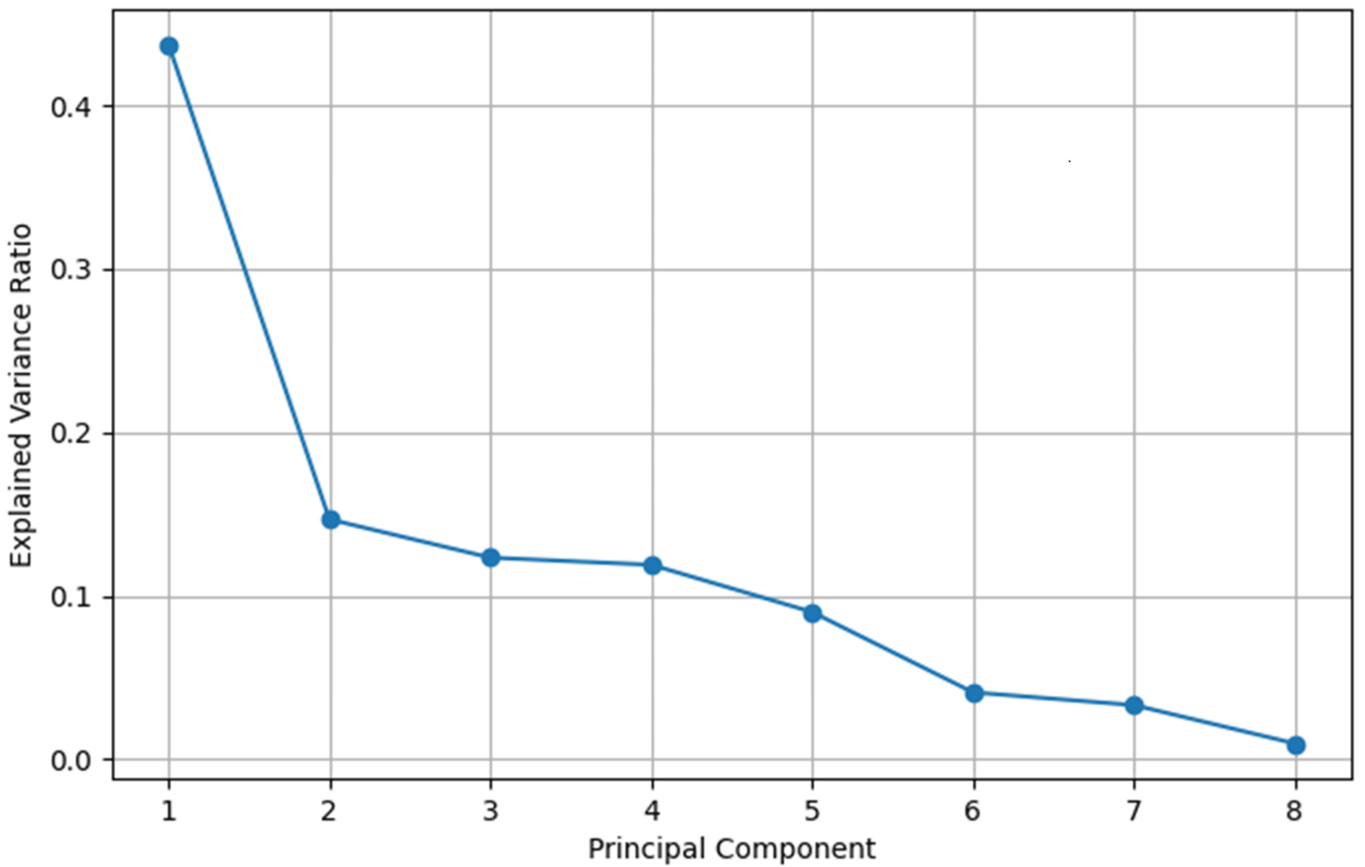
The scree plot showing variance explained by each principal component

The scree plot (Figure 6) displays the proportion of variance explained by each PC. Based on the scree plot, the first component (PC1) accounts for over 45% of the total variance in this dataset. The second component (PC2) explains a smaller, but still significant, portion. The subsequent components (PC3, PC4, etc.) explain progressively smaller amounts of variance. The elbow shape of the curve suggests that the first few components capture most of the data variability [29]. This breast cancer data study demonstrates that dimensionality can be significantly reduced by keeping only a few of the top PCs, which simplifies the data without losing valuable information.

## Discussion

Model performance comparison

**Figure 7:**
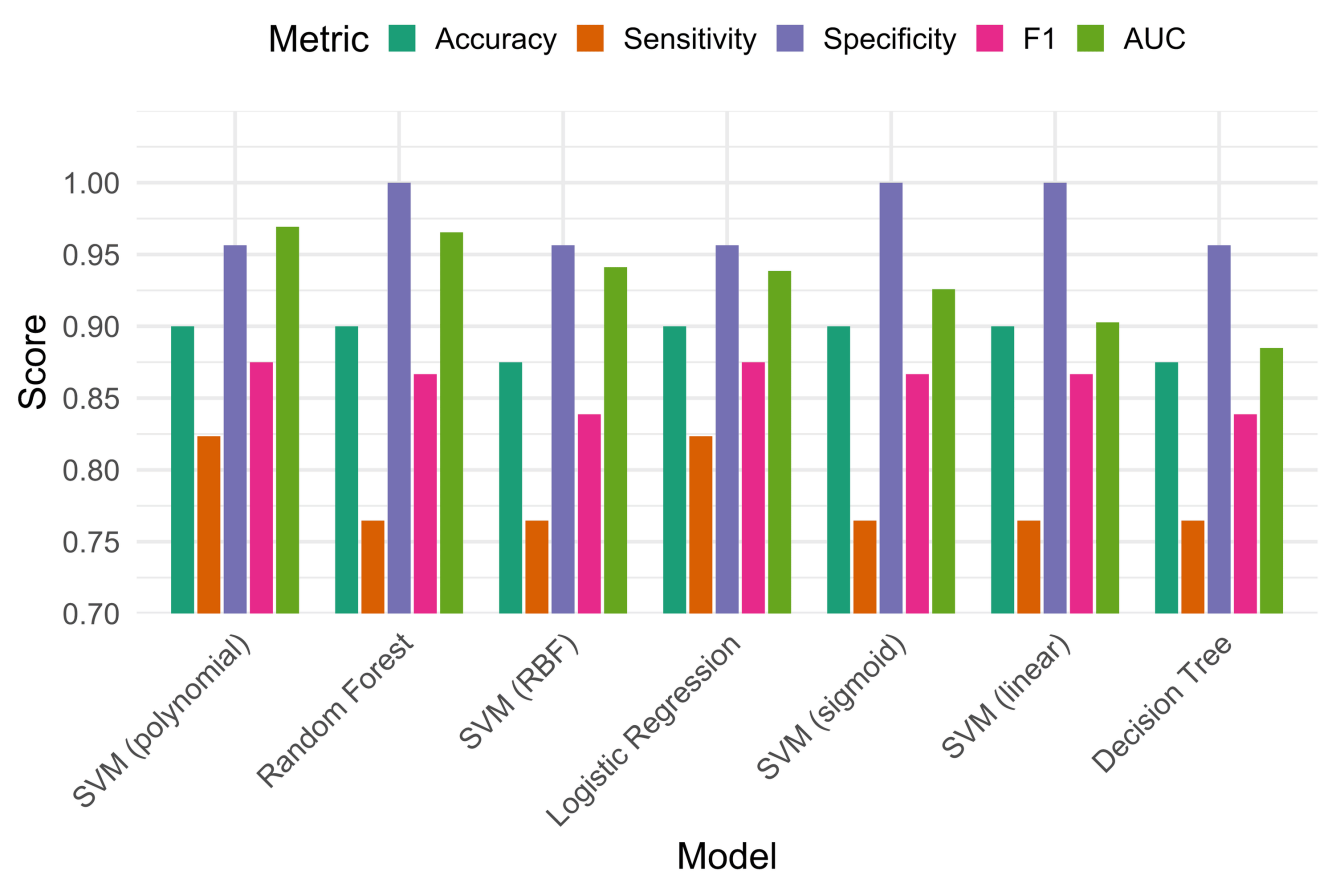
Comparison of performance metrics across all trained classification models SVM: support vector machine; RBF: radial basis function; AUC: area under the curve

This study evaluated the performance of four supervised ML algorithms, namely, logistic regression, decision tree, random forest, and SVMs with four different kernels (linear, polynomial, RBF, and sigmoid), for the classification of breast cancer tumors. All models demonstrated strong performance, achieving an accuracy greater than 87% as detailed in Table 3 and Figure 7.

**Table 3:** Comprehensive model performance comparison SVM: support vector machine; RBF: radial basis function; AUC: area under the curve

Model	Accuracy	Sensitivity	Specificity	F1 score	AUC
Logistic regression	0.900	0.8235	0.9565	0.8750	0.9386
Decision tree	0.875	0.7647	0.9556	0.8387	0.8849
Random forest	0.900	0.7647	1.0000	0.8667	0.9630
SVM (linear)	0.900	0.7647	1.0000	0.8667	0.9028
SVM (RBF)	0.875	0.7647	0.9565	0.8387	0.9412
SVM (polynomial)	0.900	0.8235	0.9565	0.8750	0.9693
SVM (sigmoid)	0.900	0.7647	1.0000	0.8667	0.9258

Logistic regression, random forest, and all SVM kernels except RBF achieved an accuracy of 90%. Among these, the polynomial SVM kernel and logistic regression yielded the highest sensitivity of 82.35%, indicating their effectiveness in correctly identifying malignant cases. As shown in Figure 3, the random forest and polynomial SVM models emerged as the most effective classifiers, achieving the highest AUC values of 96.3% and 96.93%, respectively, for distinguishing between malignant and benign tumors. The AUC metric is a robust measure of a model's diagnostic performance, representing the probability that a classifier will rank a randomly chosen positive instance higher than a randomly chosen negative one. Importantly, logistic regression not only provided high accuracy (90%) and AUC (93.86%) but also offered strong interpretability, identifying Age, Tumor Size, and Breast Quadrant as statistically significant predictors of malignancy. The analysis of tree-based models provided crucial insights into feature importance. The decision tree model identified Involved Lymph Nodes, Age, and Tumor Size as the most significant predictors. In particular, nodal status was a primary determinant (Figure 3) for classification, which aligns with clinical understanding. The random forest corroborated these findings, ranking Tumor Size and Involved Lymph Nodes as the most influential predictors, followed by Metastasis and Age (Figure 4). These results reinforce the clinical relevance of nodal status and tumor size as central determinants of breast cancer diagnosis.

**Figure 8:**
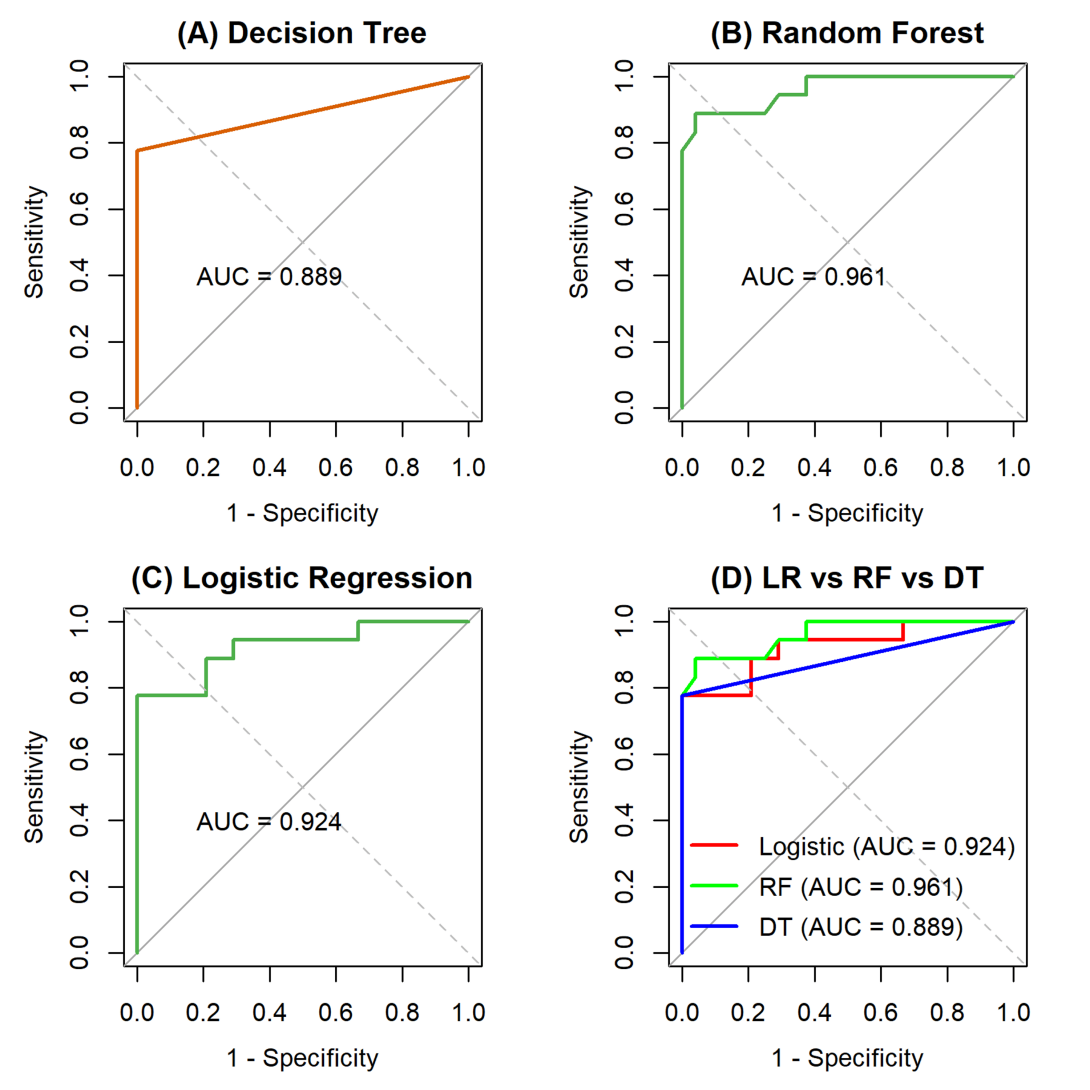
ROC curves for three models, including (A) logistic regression, (B) decision tree, and (C) random forest. (D) is for comparison of the three models ROC: receiver operating characteristic; AUC: area under the curve; RF: random forest; DT: decision tree

The ROC curve analysis further illustrated the models' diagnostic performance (Figure 8). Among the three models, random forest achieved the highest AUC (0.963), confirming its effectiveness in distinguishing malignant from benign tumors. The AUC measures the diagnostic test's ability to discriminate between patients and non-patients, representing the probability that the model will rank a randomly selected patient higher than a randomly selected non-patient [30].

**Figure 9:**
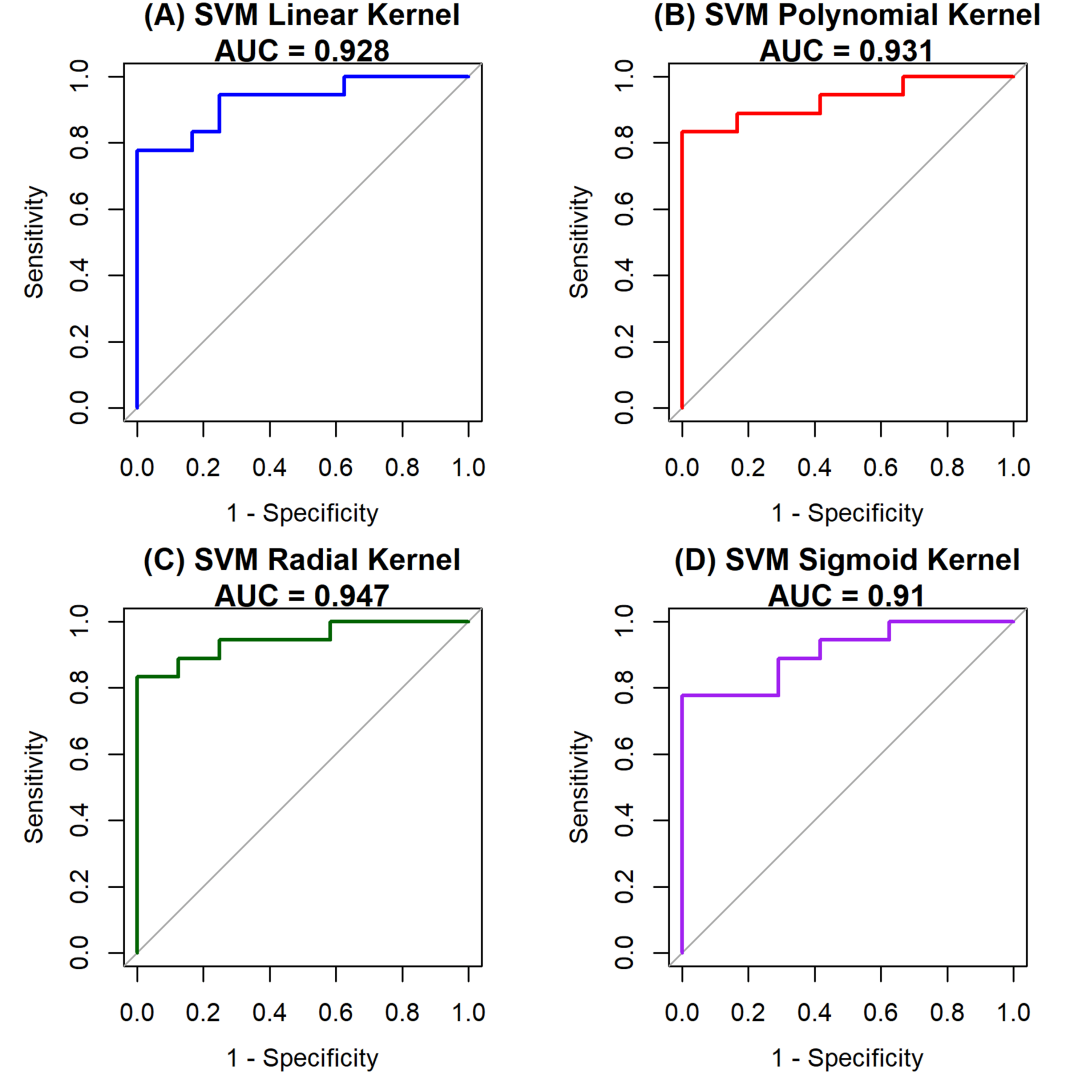
A 2×2 grid showing the individual ROC curves and AUC values for the four SVM kernels: (A) linear, (B) polynomial, (C) RBF, and (D) sigmoid ROC: receiver operating characteristic; AUC: area under the curve; SVM: support vector machine; RBF: radial basis function

For SVM models with four kernels (Figure 9), the polynomial kernel achieved the best AUC (0.969), followed by the RBF kernel (0.941), indicating that nonlinear decision boundaries provide a slight performance advantage over linear separation. Nevertheless, the excellent performance of the linear SVM (90% accuracy; AUC=0.903) and logistic regression underscores that the dataset is largely linearly separable, a finding supported by t-SNE visualization and decision boundary plots (Figure 1 and Figure 5), which revealed clear clustering of benign and malignant samples.

We also compared the SVM performance on the full dataset versus the dimensionally reduced dataset containing only the first two PCs (Table 2). The linear and RBF kernels maintained their high accuracy, validating the use of PCA for this dataset, while the polynomial SVM experienced a minor decline (from 90% to 88.3%). By contrast, the sigmoid kernel's performance decreased markedly, highlighting its instability relative to other kernels. These results demonstrate that a simplified feature representation is sufficient for robust classification when paired with appropriately chosen kernels. Overall, this comparison validates the use of PCA for this dataset and powerfully illustrates that a simplified representation is sufficient for robust classification when using appropriate kernels like linear or RBF.

We validated our analysis by running it in both R and Python. The results were highly consistent across both platforms, which is expected for a small dataset where minor software differences have a negligible effect. As shown in Table 2, the SVM models produced nearly identical accuracy scores. This confirms that our findings are robust and reflect genuine patterns in the data, rather than being an artifact of a specific software tool. In this cancer data study, we have found that in all models, Tumor Size, Involved Lymph Nodes, Metastasis, and Age consistently emerged as the most influential predictors, with tree-based models highlighting axillary lymph node involvement and regression models emphasizing the breast quadrant as additional significant variables. Collectively, these results indicate that simpler, interpretable models such as logistic regression and linear SVM are highly effective for this dataset, while more complex nonlinear models offer only marginal improvements.

Study limitation

This study has several limitations. The dataset was relatively small and sourced from a single public repository, which may limit the generalizability of the findings. The absence of important clinical variables, such as genetic markers, histopathological details, and treatment history, also limited the models' ability to capture the full complexity of breast cancer diagnosis. Furthermore, the retrospective design is susceptible to data quality issues, and the evaluation method relied on a single data split without external validation, risking overly optimistic performance estimates. Finally, minor differences observed between the R and Python implementations suggest that results may be sensitive to software defaults and parameter settings. Future studies using larger, multi-center cohorts and richer clinical data are needed to validate these findings and assess their clinical applicability.

## Conclusions

This study successfully compared the performance of logistic regression, decision tree, random forest, and SVM models for the classification of breast cancer tumors and to identify the key predictors for breast cancer diagnosis. All models demonstrated strong predictive ability, with accuracies above 87%, but logistic regression achieved the best balance of accuracy (90%) and interpretability, identifying Tumor Size, Age, and Breast Quadrant as statistically significant predictors of malignancy. Tree-based models emphasized Involved Lymph Node as an additional key factor. A detailed analysis of ROC curves revealed that random forest and polynomial SVM achieved the highest discriminatory power with AUC values of 96.3% and 96.93%, respectively. This work also validates the effectiveness of PCA as a dimensionality reduction technique. Consistently across all models, Tumor Size and Age emerged as the most influential features and provide a robust benchmark for future clinical applications and predictive performance.
